# Resetting a functional G1 nucleus after mitosis

**DOI:** 10.1007/s00412-015-0561-6

**Published:** 2016-01-04

**Authors:** Ines J. de Castro, Ezgi Gokhan, Paola Vagnarelli

**Affiliations:** College of Health and Life Science, Research Institute of Environment Health and Society, Brunel University London, Uxbridge, UB8 3PH UK

**Keywords:** Phosphatases, Mitotic exit, Chromatin, Nuclear envelope, Cell division

## Abstract

The maintenance of the correct cellular information goes beyond the simple transmission of an intact genetic code from one generation to the next. Epigenetic changes, topological cues and correct protein-protein interactions need to be re-established after each cell division to allow the next cell cycle to resume in the correct regulated manner. This process begins with mitotic exit and re-sets all the changes that occurred during mitosis thus restoring a functional G1 nucleus in preparation for the next cell cycle. Mitotic exit is triggered by inactivation of mitotic kinases and the reversal of their phosphorylation activities on many cellular components, from nuclear lamina to transcription factors and chromatin itself. To reverse all these phosphorylations, phosphatases act during mitotic exit in a timely and spatially controlled manner directing the events that lead to a functional G1 nucleus. In this review, we will summarise the recent developments on the control of phosphatases and their known substrates during mitotic exit, and the key steps that control the restoration of chromatin status, nuclear envelope reassembly and nuclear body re-organisation. Although pivotal work has been conducted in this area in yeast, due to differences between the mitotic exit network between yeast and vertebrates, we will mainly concentrate on the vertebrate system.

## Phosphatases at mitotic exit: who is tidying up what after the mitotic party

CDK1-cyclin B activity is crucial for mitotic entry, and its inhibition promotes mitotic exit. The APC/C-Cdc20 complex timely degrades the mitotic cyclins and promotes mitotic exit through CDK down-regulation. Although this represents a crucial event for mitotic exit, dephosphorylation of CDK1 substrates is an essential step, and phosphatases take control of the transition progression (Bollen et al. 2009; Grallert et al. 2015; Mochida and Hunt 2012). In view of the events that characterise mitotic exit, activation and localisation of these phosphatases becomes a key control step for the reformation of a functional G1 nucleus.

In vertebrates, PP1 and PP2A have emerged as the most important phosphatases for the regulation of mitotic exit. Most PP1 complexes contain one catalytic and one regulatory subunit, where the interaction between the subunits typically involves short docking motifs. In vertebrates, almost 200 interacting proteins have been identified in this process, and they function as inhibitors of the catalytic activity, substrate-specifying subunits, targeting subunits or substrates. PP1 has also three isoforms (alpha, beta and gamma), and all these isoforms appear to have specific roles in the cell cycle (Trinkle-Mulcahy et al. 2001). Some targeting subunits have preference for one of the isoforms but this specificity is still not very well understood.

PP2A has a catalytic subunit (C), a scaffolding subunit (A) and most of the complexes also contain a variable subunit (B) that acts as a substrate specifier. The B subunits are B55, B56 and PR72, and they have different isoforms (Hunt 2013; Kurimchak and Grana 2012).

Studies on the identification of phosphatases that control mitotic exit have suggested not only that both PP1 (Wu et al. 2009) and PP2A (Schmitz et al. 2010; Mochida et al. 2009) play an essential role in resetting the new G1 nucleus but that they required to be re-activated at anaphase onset for a proper execution of late mitotic events (Skoufias et al. 2007). In fact a form of PP1, PP1 alpha, is inhibited during mitosis by CDK phosphorylation on Thr 320 (Dohadwala et al. 1994), as is PP2A (Mochida et al. 2009; Gharbi-Ayachi et al. 2010). A recent study in fission yeast has shown the existence of an interplay between PP1 and PP2A phosphatases at the metaphase/anaphase transition. Grallert and co-workers revealed that in early mitosis both PP2A/B55 and PP2A/B56 are phosphorylated and bound to phosphorylated PP1. This appears to lock these two major phosphatases in their inactive states. At the transition from mitosis to anaphase, CDK inactivation allows PP1 activation (by auto-dephosphorylation) and dephosphorylation of the bound PP2A/B55, which is consequently released and activated. The activated PP2A/B55 then dephosphorylates PP2A/B56 when PLK1 (the counteracting kinase for B56) activity decreases towards the end of mitotic exit. The dephosphorylation of the PP1 binding site on PP2A/B56 allows recruitment of PP1, which in turn activates the former, resulting in the full activation of PP1 and PP2A complexes (Grallert et al. 2015).

Although still not quite water-tight, this sequential removal of inhibitory signals can secure a correct progression of mitotic exit where some events need to precede others in order to complete the reformation of G1 (Bollen 2015). In a way, these cascades can be defined as a molecular clock of the system (see later in the review).

**Fig. 1:**
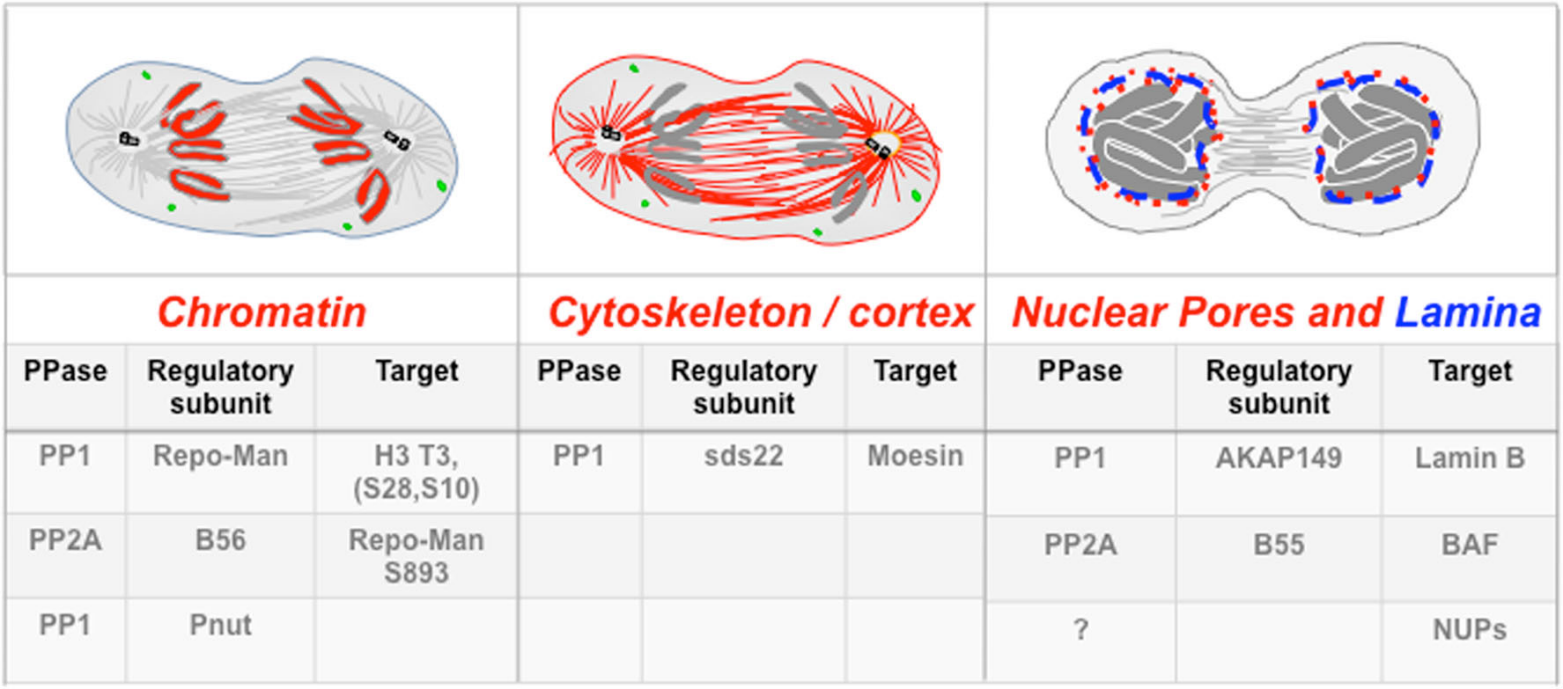
Phosphatases involved in G1 re-organisation. See text for details

Figure 1 lists the known substrates that have so far been identified in mitotic exit. It clearly shows that most of the processes lack a dedicated phosphatase and a lot of work is still required to complete the picture and to identify all the key players.

Given the importance of PP1 and PP2A in mitotic exit and the recent knowledge that we have acquired on their substrate specificity, it will become important in the near future to identify their specific mitotic substrates in space and time to understand the orderly progression of events that allow the reformation of a functional nucleus after mitosis.

## Re-establishing the nuclear barrier: nuclear envelope reassembly

The nuclear envelope provides the physical separation of the DNA from the cytosol. It comprises an inner nuclear lamina, containing a nuclear and luminal layer packed with a wide variety of proteins such as lamina filaments (B- and A-type lamins), LBR, emerin and other lamina-associated proteins (LAP1/LAP2). It is at the inner nuclear membrane (INM) that chromatin binding proteins such as BAF and HP1 (heterochromatin protein 1) provide a bridge between the lamina and chromatin. The outer lamina extends to the endoplasmic reticulum (ER) and into the cytoplasm. Intercalating open spaces in the nuclear envelope (NE) are the nuclear pore complexes (NPC). These provide an open structure for rapid transport of proteins and transcripts into and out of the nucleus. The central core of the NPC is the Nup107–160 complex; the nucleoporins Nup153, Nup50 or ELYS are located on the chromatin side, whereas the cytoplasmic NPC is characterised by the presence of Nup214, Nup88 and Nup358 (Bernad et al. 2004).

During open mitosis, which is the case in mammalian systems, the nucleus goes through major transformations. One is the physical tearing of the nuclear envelope (nuclear envelope break-down (NEBD)), which allows separation of sister chromatids after chromosomes have condensed. This process is reversed shortly afterwards (within 30/90 min after NEBD), and the new G1 nucleus re-organises to its original state.

Several kinases play a major role in NEBD. Dissociation of chromatin from the nuclear envelope occurs through phosphorylation events on lamins (A/C and B) and lamina-chromatin-associated proteins. The first V-type filaments that disappear from the rim are the A-type lamins in early prophase, a target of CDK1 (Peter et al. 1990). In late prophase/early prometaphase, protein kinase C activity then leads to the dissociation of B-type lamins from the rim (Heald and McKeon 1990). However, a pool of lamin B1 and LAP2 alpha appears to be retained on chromatin during mitosis (Martin et al. 2010). The NPC dissociates via post translational modifications of several nucleoporins where the key event appears to be the phosphorylation of the peripheral Nup98 by various kinases (Laurell et al. 2011).

Once phosphorylated, some NE proteins accumulate in the tubular network of mitotic ER (Puhka et al. 2007; Ellenberg et al. 1997), others associate with the mitotic spindle playing an important role in spindle formation (Harel and Forbes 2004; Nachury et al. 2001) and some sub-complexes associate with kinetochores (Nup107–160; Platani et al. 2009; Loiodice et al. 2004).

The reformation of the nuclear envelope (the reversal of the process described above) requires the re-association of all the components that were disassembled during mitosis in a sequential and timely fashion, and therefore, it is also a highly regulated mechanism. Protein phosphatase 1 is recruited to the periphery of chromosomes at telophase directing dephosphorylation of lamin B and promoting its polymerisation (Thompson et al. 1997). Although lamin A is thought to bind chromatin at a later stage, after the NPC is assembled (Moir et al. 2000), live-cell imaging analyses allowed the visualisation in early anaphase of a small pool of lamin A at specific chromosome regions, together with BAF, emerin and LAP2alpha, from where it extends at later stages (Haraguchi et al. 2008; Dechat et al. 2004). Therefore, it appears that lamin B and lamin A accumulation follow different pathways that still need to be determined in their molecular details.

NPC formation also follows timely regulated mechanisms. The Nup107–160 is the first to associate to chromatin via the AT-hook repeats of ELYS, and, following this, other nucleoporins such as Nup93, Nup98 or Nup62 are recruited (reviewed in (Guttinger et al. 2009)). To give some idea of the timings involved, Nup62 associates 3 min after the accumulation of Nup107–Nup160 (Lu et al. 2011).

The accumulation of a subset of nucleoporins such as Nup153 is also regulated by RanGTP (Rasala et al. 2008); these NPC components are in fact sequestered by importin-β during early mitosis (Harel et al. 2003), and, at anaphase, RanGTP reverses the binding by allowing NPC dissociation from the importin β complex and deposition around the anaphase chromatin. However, other components such as importin β binding (IBB) domain do not re-associate with the reforming NE until late telophase (Lu et al. 2011).

This sequentiality of events during NE reformation suggests that an orderly dephosphorylation of key proteins occurs to allow macromolecular complexes to form and target to the right place at the right time. These could be dictated either by activation of different phosphatases at different times during mitotic exit (as we discussed before) or by the position of the segregating chromatin within the anaphase cell or a combination of both of the above. A topological control of the process appears to occur in drosophila where the position of the chromatin seems to allow or prevent reformation of the NE during mitotic exit (Afonso et al. 2014). This control mechanism appears to be regulated negatively by Aurora B and positively by PP1/PP2A. However, the specific phosphatase complexes and the substrates involved are still elusive, and only a handful of phosphatases have been identified thus far playing a role in NE reformation: PP1/Repo-Man, PP2A and PP1/AKAP149 (Fig. 1). The PP1 targeting subunit AKAP149 was shown to dephosphorylate B-type lamins by anchoring PP1 at the NE throughout interphase, and premature disruption of this complex results in intranuclear lamina solubilisation (Steen et al. 2000). Another PP1 targeting subunit, Repo-Man binds directly and dephosphorylates importin β, and targets it to the reforming NE in early anaphase (Vagnarelli et al. 2011) (Vagnarelli and Earnshaw 2012) together with Nup153 (de Castro et al., in preparation). Although probable, it is still not clear if this complex is also involved in the dephosphorylation of these nuclear components during anaphase. On the other hand, BAF is dephosphorylated by PP2A facilitating its re-association with chromatin. BAF is phosphorylated by VRK-1 kinase in mitosis, and LEM blocks VRK activity during mitotic exit. It is this fine crosstalk between stages of phosphorylation and dephosphorylation that aid NEBD and reassembly, respectively (Asencio et al. 2012). However, considering the number of NE components that are phosphorylated during mitosis by multiple kinases, it is unlikely that the whole NE reassembly process can be controlled with just these few phosphatases.

## Ensuring chromatin function after mitosis

### Epigenetics in mitosis

In the interphase nucleus, several levels of organisation control chromatin function. Chromatin structure (condensation/decondensation), histone modifications, transcriptional machinery interactions and nuclear bodies are all required to ensure proper gene expression programmes. Here, we will discuss how these processes are controlled during the passage throughout mitosis.

**Fig. 2:**
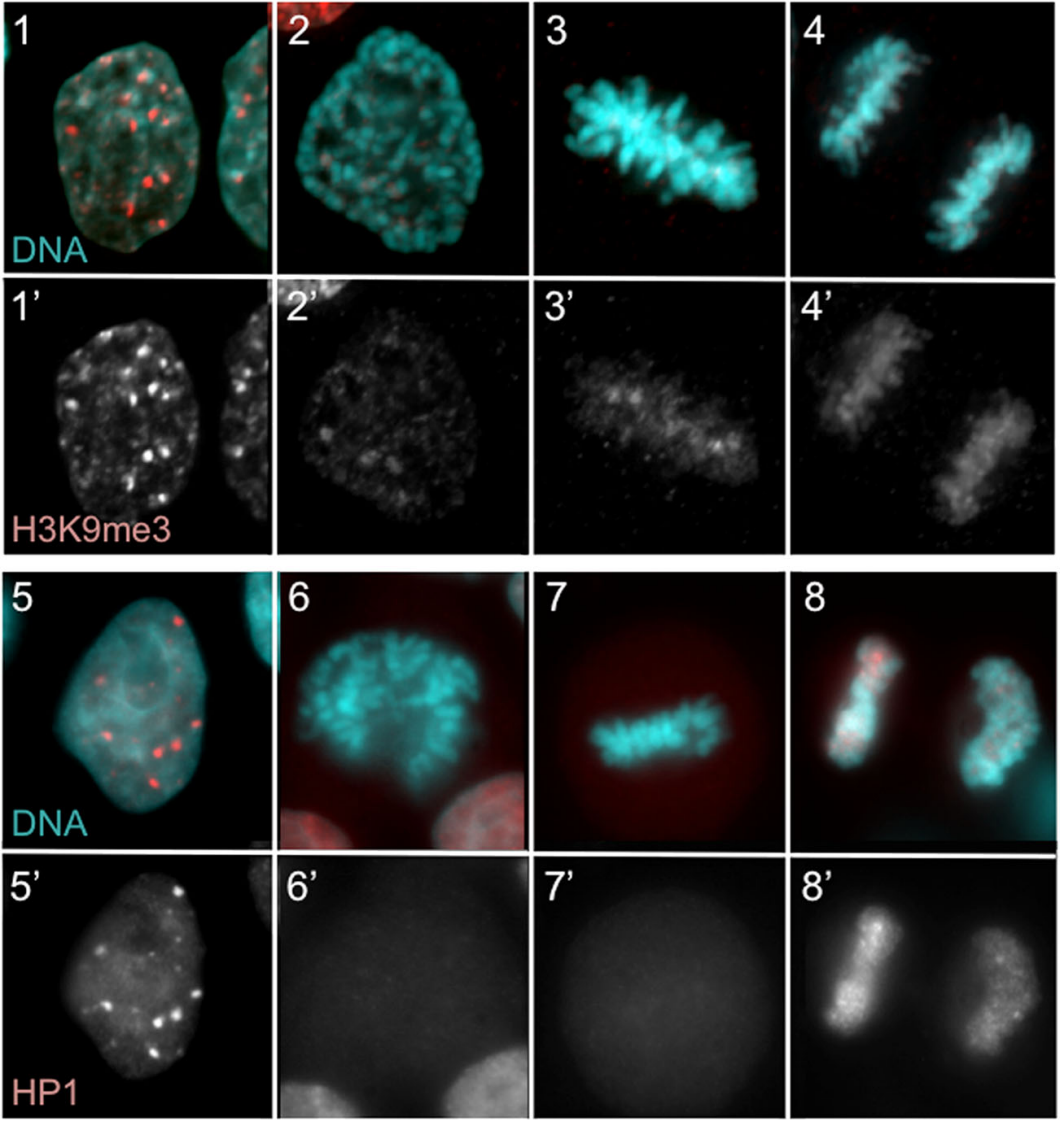
Phospho-switches in chromatin re-organisation after mitosis. H3K9me3 (**1**–**4**) is the docking site for HP1 binding (**5**–**8**). In mitosis, H3S10 becomes phosphorylated by Aurora B kinase. This phosphorylation masks the H3K9me3 epitope for antibody recognition in prophase (**2**) and metaphase (**3**) but also blocks HP1 from binding (**6** and **7**). During mitotic exit, the removal of H3S10 phosphorylation by PP1/Repo-Man allows HP1 to target to the chromatin and re-establish the specific chromatin domains (**4**, **8**)

Mitotic chromatin condensation is a complex process that involves changes both in chromatin compaction and organisation. It is achieved by modification of both histone (Wilkins et al. 2014) and non-histone proteins (Vagnarelli and Earnshaw 2012). Some of these modifications are directly linked to condensation while others mediate a temporal switch that releases/attracts specific protein(s) to chromatin. One of the landmark changes in mitotic chromatin is represented by histone H3 phosphorylation by Aurora B and haspin kinase. Aurora B phosphorylates H3 at Ser10, and this modification leads to dissociation of HP1 from the neighbouring H3K9me3 (Fig. 2). Accumulation of HP1 at H3K9me3 sites in interphase is a well-studied mark for gene repression. Recently, it was shown in *S. cerevisiae* that H3S10ph also leads to deacetylation of H4 thus enhancing the condensed chromatin status (Wilkins et al. 2014). However, in vertebrates, lack of mitotic H3S10 phosphorylation does not affect chromosome compaction or structure (Xu et al. 2009). H3S28 is also phosphorylated in mitosis. Once again, the K27 lysine that follows S28 is subject to post-translation modifications (PTMs); for example, the repressive polycomb group of proteins target H3K27 for methylation but phosphorylation of S28 displaces polycomb from H3K27, which then can be targeted by acetylases (Lau and Cheung 2011). Although this mechanism is quite well described in interphase, it remains to be elucidated whether the same is true in mitosis.

H3 is also phosphorylated at T3 by haspin kinase in mitosis (Wang et al. 2010). This phosphorylation, besides controlling the targeting of the chromosome passenger complex, also produces the dissociation of the transcription factor TAF3 from the histone mark H3K4me3, once again reverting target genes into a repressed state (Varier et al. 2010). The vast majority of PTMs are maintained through mitosis, ensuring propagation of a specific epigenetic status to daughter cells. H3K9 is methylated throughout mitosis (Fischle et al. 2005), and although a fraction of Suv39 (the H3K9 methyalse) accumulates at centromeres at prometaphase, the majority remains dissociated until after the metaphase to anaphase transition (Aagaard et al. 2000). The nearby S10 phosphorylation might have led to the masking of the former epitope during mitosis which in the past has generated confusing statements about the presence/absence of these modifications in mitosis (Fig. 2). Concomitantly, H3K27me3 persists at similar levels through mitosis (Zee et al. 2012; Hansen et al. 2008; Hansen and Helin 2009; Follmer et al. 2012) but association with the polycomb group of proteins (PcG) at the vast majority of target sites is lost. This being the general rule, there are exceptions where some genes remain associated with PcG throughout mitosis (Follmer et al. 2012). Similarly, the histone variant H2A.Z is maintained during mitosis, where it is preferentially found at chromatin sites that will become active genes or genes poised for activation (Kelly et al. 2010). Histone acetylation H3K27ac and H3K9ac are also maintained throughout mitosis. However, studies have shown that histone acetyltransferases and deacetylases dissociate from chromatin at early mitotic stages re-localising at late mitosis (Kruhlak et al. 2001). Interestingly, H3S10 can also be O-GlcNAcylated; this is thought to be important for the maintenance of a repressive chromatin state and, since this modification persists during mitosis, could represent another bookmarking event for the next G1 (Zhang et al. 2011). Positive histone marks, H3K4 methylation (mono, di, tri), H3K79 dimethylation, H3 and H4 acetylation, are also present throughout mitosis in HepG2 cells, suggesting that positive sites of transcription are inherited and maintained during the mitotic cycle (Kouskouti and Talianidis 2005; Zhao et al. 2011).

In conclusion, there is a mitotic histone code that prepares chromatin for interphase, ensuring propagation of gene expression programmes; these states of chromatin are inherited and a binary phospho-methyl switch code ensures that the specific epigenetic readers or writers are recruited to the same places after the wave of mitotic phosphorylation is over.

So what reverts the switch during mitotic exit?

PP1/Repo-Man complex has been shown to remove H3T3ph (Vagnarelli et al. 2011; Qian et al. 2011), and we have identified Repo-Man as the phosphatase that removes H3S10ph as well (Vagnarelli et al. 2011). Repo-Man association with chromatin is dependent on the inactivation of mitotic kinase CDK1 and the dephosphorylation by PP2A (Qian et al. 2013). Association of Repo-Man with chromatin in anaphase leads to dephosphorylation of H3 through PP1. Removal of H3S10ph represents the phospho-methyl switch that is associated with HP1 binding to chromatin (Vagnarelli et al. 2011). However, more research will be required to understand which phosphatase is responsible for removing the S28ph, necessary to recruit the PRC complex to the chromatin, and other phosphatases could be involved at different chromatin sites to erase the phosphorylation marks from other histones.

### Transcription factors

Structural conformation of chromatin during mitosis is not compatible with the association of most chromatin binding proteins. Additionally, some proteins are targets for mitotic kinases, and the phosphorylated forms have less affinity for their specific chromatin (for example, Ezh2 when phosphorylated by CDK1 (Wu and Zhang 2011). Mitotic phosphorylations of histones can also play a role in weakening the interaction between chromatin and transcription factors; one example is H3T3 phosphorylation by haspin during mitosis that generates an interesting crosstalk with the adjacent H3K4me3. H3K4me3-marked promoters interact with a wide range of transcription activators. The pre-initiation complex (PIC) includes a number of general transcription factors (TFIIA/B/D/E/F/H) and RNA Pol II (RNAPII). The presence of H3T3 is inhibitory of the TFIID association with the neighbouring H3K4me3, thus resulting in repression of genes (Varier et al. 2010).

During mitosis, some active loci maintain the association with TATA-binding protein (TBP) (a subunit of TFIID and the building block of the PIC, from which other subunits are recruited). Mitotic TBP complexes seem to contain phosphatase activity (PP2A) necessary to prevent the condensation of chromatin via the local dephosphorylation of condensin. However, as a general theme, dissociation of transcription activators from chromatin sites is the most common situation, and, although it remains unknown why some genes are kept under the regulation of transcription factors, it is tempting to suggest that this allows genes to be in a potentially active state ready for transcription upon mitotic exit.

BRD4 is another transcription-associated protein that recruits the positive transcription elongation factor b (pTEFb) complex for elongation of RNAPII through phosphorylation past the transcription start site (TSS) and onto the coding region of a gene. BRD4 has been shown to associate with mitotic chromatin in some (mouse C127, NIH3T3) but not all cell lines (HeLa) (Yang et al. 2008) (Dey et al. 2009). In U2OS cells, BRD4 recruitment to post-mitotic chromatin, possibly docking on H4K5ac, precedes pTEFb and RNAPII recruitment and suggests a more global role for BRD4 in post-mitotic gene activation (Zhao et al. 2011). In a similar manner, BRD4 associates with chromatin in telophase in HeLa cells and is responsible for the recruitment of the pTEFb complex, prior to the NE assembly, suggesting priming of genes for rapid activation (Yang et al. 2008).

Apart from general transcription factors, other tissue-specific or pathway-specific transcription factors might also remain associated with chromatin during mitosis. The best understood are GATA1 and RUNX1 or FOXA1, all committed in tissue specific functions that are readily activated in G1. For example, destruction of GATA1 during mitosis was found to delay the expression of GATA1 target genes (reviewed in (Kadauke et al. 2012)). At specific sites, polycomb groups of proteins are also retained on chromatin in drosophila (Follmer et al. 2012), and interestingly, these sites demarcate boundary regions by associations with insulator binding sites (CTCF, BEAF, CP190, Chromator) that may be important to organise polycomb-regulated regions in interphase. Chromatin modifiers, such as HDACs, are also subjected to phosphorylation by Aurora B in mitosis, thereby releasing them from the repressive chromatin-associated complex NCoR (Guise et al. 2012).

Overall, the emerging picture is that the vast majority of genes are free from transcription regulators during mitosis; however, the information is maintained for ready-to-go in the next G1. Kadauke et al. dedicated a review to this specific subject (Kadauke and Blobel 2013).

### Transcription and translation

Recent research has been challenging the dogma that transcription and translation cease in mitosis. As previously mentioned, the vast majority of transcription factors are released from chromatin upon mitotic entry. In general, RNAPII rapidly re-associates with gene promoters in telophase in a timely regulated process; RNAPII initially accumulates in its initiation form together with RNAPII-associated transcription factors. Later, the elongation form of RNAPII accumulates together with the pre-mRNA processing machinery.

Although transcription is generally suppressed during mitosis, there are some exceptions; the mitotic kinase cyclin B1 is transcriptionally active during mitosis, concomitant with the fact that some transcription factors remain associated with their targets during mitosis. The synthesis of cyclin B during mitosis is apparently important for mitotic functions such as spindle assembly (Mena et al. 2010). Another example is the transcription of the centromeric α-satellite, which seems to be essential for the proper functioning of the mitotic kinetochore (Chan et al. 2012).

One of the emerging aspects is that regulation of protein levels during mitosis heavily relies on translational control. Using metabolic labelling, combined with ribosome profiling and drug-free synchronisation protocols, Tanenbaum and colleagues have identified two distinct translational programmes that occur during mitosis: (1) ∼35 % global translational repression of the bulk of mRNAs and (2) ∼200 of mRNAs that show large gene-specific changes in their translation efficiency during mitosis. This latter group encompasses mRNAs in which translation is paused during mitotic entry and resumed upon mitotic exit. An example is that of Emi1, a gene involved in inactivating the APC, which is reduced to very low levels during mitosis in order to allow the activation of APC protein and progression of the cell cycle. When cells exit mitosis, translation of Emi1 is quickly activated. The advantage of this mechanism is the ability of regulating protein levels in a very short period of time compared to transcription, and its rapid reversibility enables protein synthesis to restart quickly when cells exit from mitosis and enter G1 (Tanenbaum et al. 2015).

## Resetting the nuclear topology: nuclear bodies and chromosome territories

### Nucleolus, nuclear specles, cajal bodies and PML bodies

The vast majority of nuclear bodies are disassembled during mitosis. However, some may retain mitotic sub-complexes that might help the reassembly process. Here, we will briefly mention the re-organisation of nuclear bodies during mitosis but for detailed reviews refer to (Dundr and Misteli 2010; Mao et al. 2011). Several RNAPI transcription factors as well as nucleolar processing proteins are phosphorylated by mitotic kinases. This includes phospho-regulation of the nucleolar protein Ki-67 (Booth et al. 2014). The disassembly of the nucleolus results in the dissemination of processing machinery factors and unprocessed rRNAs in nucleolus-derived foci (NDF) or association with the perichromosomal layer, a mitotic chromosome compartment assembled by Ki-67 (Booth et al. 2014). In telophase, upon activation of rDNA transcription, the RNA processing machinery starts accumulating at nucleolar organiser regions (NORs), forming the prenucleolar bodies, together with the remains of NDFs. Therefore, the RNA processing machinery progresses from one cell cycle to the next. Recent advances demonstrate that Ki-67, recently identified as another PP1 targeting subunit and substrate (Booth et al. 2014; Takagi et al. 2014), is responsible for the correct re-assembly of the nucleolus in G1.

Nuclear speckles, or splicing speckles, are punctuating foci where splicing factors accumulate for efficient RNA processing events. When splicing speckles disassemble, its machinery remains dispersed through mitosis aggregating in mitotic inter-chromatin granules (MIGs). In early G1, when RNAPII transcription is re-activated, pre-mRNA splicing machinery starts accumulating in the nucleus, including the migration of MIGs (Spector and Lamond 2011).

Cajal bodies (CB) accumulate small nuclear ribonucleoproteins (snRNP), SMN and coilin. They remain associated in mitosis in so-called mitotic cajal bodies (MCB). Upon reformation of the nuclei, MCBs disintegrate, and the components, first coilin and then SMN, start accumulating in the nucleoplasm for the new formation of CB (Sleeman et al. 2001).

Promyelocytic leukemia (PML) bodies are thought to regulate post translational processes as they partner with SUMO and ubiquitin ligases. They have also been associated with translocations in cancer. PML bodies remain aggregated during mitosis in mitotic accumulations of the PML protein (MAPPs). Reformation of this nuclear body includes transition from the cytoplasm of its main components, namely SP100 and DAXX (Dellaire et al. 2006).

### Chromosome territories

Once the nucleus has reformed, spatial regulation of chromatin becomes extremely important. Although much progress has been made on the different processes that take the compact mitotic chromosomes back to the interphase chromatin state, there are still a few unanswered questions.

It is well established that chromosomes after mitosis are arranged in territories in a non-random manner (Croft et al. 1999). The higher order of chromatin organisation has been a main focus of the field in recent years, and many progresses have been made thanks to a wide variety of approaches (Fraser et al. 2015; Wilson and Weis 2015). Chromosome territories (CTs) are disassembled during the cell cycle upon condensation of chromatin in early mitosis. Chromosome re-organisation starts in prophase with individualisation into rod-like structures by the action of condensins (Hudson et al. 2003). Condensin II is present in the nucleus during interphase whereas condensin I enters only to assist in the condensation of mitotic chromatin after NEBD. Later on, both condensin complexes play fundamental roles in the segregation of sister chromatids. The position that chromosomes occupy in the next G1 nucleus seems to be dependent on condensin II (Bauer et al. 2012; Joyce et al. 2012). The predictive positions that each chromosome will occupy in the nucleus appear related to its position on the metaphase plate, when chromosomes attach the mitotic spindle and line up to prepare for moving to the opposite poles. Because this positioning is not overall conserved in mitosis, the locations of the CTs do not seem to be propagated from mother to daughter cells; however, two sister cells are likely to have similar CTs organisation (Orlova et al. 2012). A locus can thus occupy different positions in proximity to other nuclear compartments after mitosis and therefore relocate from the periphery to the nucleolus in one cell division (Chubb and Bickmore 2003). After their establishment, CTs distribution, volume and morphology are relatively confined during the interphase (Walter et al. 2003) (Muller et al. 2010) although local movements occur by looping out events. In support of this line of evidence, new developments of the DamID technique have shown that lamina-associated domains (LADs) interacting chromatin do not entirely preserve their location after mitosis. By tagging the methylation of GATC sequences generated by Dam when fused to Lamin B1, Kind and colleagues were able to follow lamina-associated domains throughout the cell cycle. LADs at the daughter cells were found to be associated with nucleoporin-associated regions (NARs), suggesting that a different mechanism of repression might be acting at these chromosome loci. Interestingly, chromatin association with lamina depends on H3K9me2 and its methylase G9a, suggesting that the peripheral lamina positioning mechanism is more akin to be directed by epigenetics (Kind et al. 2013) (Kind and van Steensel 2014). Indeed, double knockout of lamins B1 and B2 does not alter chromatin-lamina interactions in the permissive mES cells when using emerin as a reader of LADs (Amendola and van Steensel 2015). Additionally, the authors have shown that knockdown of lamin A/C in the double knockout mutant cell line retains LADs, suggesting that other tethering mechanisms might be involved in the lamina/chromatin interaction.

All the evidence so far obtained in different systems indicates that some level of “location bookmarking” might exist within loci. Using synthetic transcription factors, Therizols and colleagues were able to activate Ptn in ES cells and induce its movement from the periphery to the centre of the nucleus. Interestingly, this location was retained even 7 days after the absence of stimuli albeit the locus was then transcriptionally inactive (Therizols et al. 2014).

In eukaryotes, topologically associating domains (TADs) divide compartments into nuclear subdomain containing clusters of multiple regulatory elements tethered by long-range interactions (Gibcus and Dekker 2013). TADs are largely dissociated during mitosis (Naumova et al. 2013). It still remains to be determined how in G1 TADs are re-established and how the loading of complexes at boundaries are formed for proper organisation. This could be mediated by bookmarking (epigenetic or epigenetic readers) that is maintained during mitosis. Assembly of these higher order structures could be mediated by transcription factor complexes bound to chromatin, or preferential clustering of chromatin domains that are similar in their histone modifications. This is consistent with the observations previously mentioned that patterns of several histone modifications are cell type-specific and are maintained in mitotic chromosomes. This is a testable hypothesis that predicts a specific order of events in early G1 with specific roles for DNA elements and protein machineries.

## Regulation of the process in space and time

### Clocks, gradients and forces

From what has been discussed previously, it appears quite evident that the reassembly of the G1 nucleus is controlled in space and time. In order to achieve this four-dimensional regulation, there must be cellular cues that control a progression of events based on the time after commitment to division, positions in the 3D space of the anaphase cell and balance of forces that organise and direct the newly reforming structures. Recent developments in the analyses of mitotic exit have led to the identification of cellular clocks, gradients and mechanical forces that contribute to the execution of late mitotic events and have allowed a start to unravelling the complex picture of mitotic exit execution and G1 nucleus organisation.

### Molecular clocks

The first major advancement towards our understanding of mitotic exit execution came from studies in budding yeast. In this experimental system, mitotic exit execution exquisitely depends on CDK down-regulation and Cdc14 phosphatase activation (Culotti and Hartwell 1971; Noton and Diffley 2000; Surana et al. 1993). Although a balance between decreasing CDK and increasing cdc14 activities can explain an ON/OFF transition state, it does not explain the sequential nature or order of different events. For example, both early mitotic exit events like spindle elongation and late events such as spindle disassembly are regulated by cdc14 activity but why do they occur at different times?

In both human and budding yeast, expression of indestructible mitotic cyclins block mitotic exit in a dose-dependent manner at sequential steps suggesting the existence of a threshold for the phosphorylation of different substrates in mitosis. This concept was demonstrated to be correct by using a FRET-based biosensor to measure cyclin B1-CDK1 activity and the timing of occurrence of mitotic events (Gavet and Pines 2010). The identification of cyclin B mitotic interactors has corroborated this piece of data and identified key components in the process (Pagliuca et al. 2011). However, inactivation of CDK1 alone is not sufficient to drive mitotic exit, and activation of CDK1 counteracting phosphatases is also required in all organisms studied so far.

To gain a better understanding of mitotic exit regulation, it will be important to obtain a map of sequential dephosphorylation events in space and time. A step in this direction has been undertaken in budding yeast where the dephosphorylation timing of a series of well-characterised CDK substrates during mitotic exit was analysed (Bouchoux and Uhlmann 2011). In this system, an ordered dephosphorylation of mitotic CDK substrates with a timing matching their expected roles was observed. The sequential order could be explained by cdc14 phosphatase having different affinities for the substrates where higher catalytic efficiencies of cdc14 are observed for its early targets. CDK substrates whose dephosphorylation contributes to chromosome segregation and anaphase spindle elongation were dephosphorylated early, before substrates implicated in spindle disassembly, replication origin relicensing, and return of the cell cycle to G1. This could easily provide an explanation on how quantitative changes of the phosphatase-to-kinase ratio over the course of mitotic exit instruct substrate dephosphorylation at sequential thresholds.

Marked differences in the timing of CDK substrate dephosphorylation have been observed in vertebrates (Mochida et al. 2009). Therefore, sequential CDK substrate dephosphorylation under the control of phosphatase-to-kinase thresholds operates in most eukaryotes and constitutes a conserved aspect of cell cycle regulation. These biochemical switches become very important at the M/G1 boundary. The biochemical clock of mitotic exit can explain well the sequential order of events, and possibly, it is the necessary and only sufficient requirement for mitotic exit in a test tube; however, within the cells, several substrates are linked to structures (no free diffusion. Therefore, other features need to be considered in modelling mitotic exit in the four-dimensional space.

### Molecular gradients

**Fig. 3:**
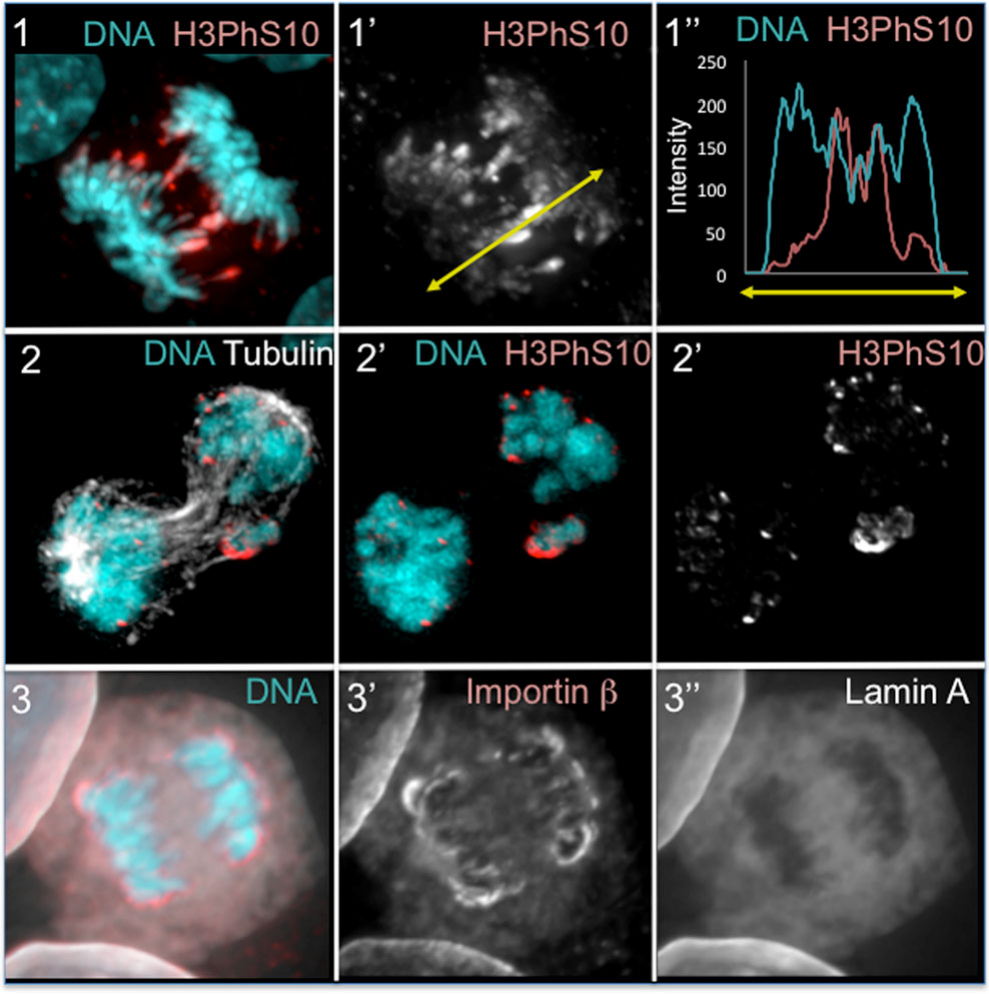
Molecular gradients in anaphase control the spatial reassembly of nuclear structures after mitosis. **1**–**2** Dephosphorylation of histone H3 (T3, S10 and S28) starts from the pole-ward side of the segregating chromatids (**1**,**2**). The chromatin that is still in the midzone presents high phosphorylation compared to the chromatin at the poles generating a gradient (**1”**). Aurora B activity in the midzone/midbody coupled to the absence of the H3 counteracting phosphatase activity (Repo-Man/PP1) on the lagging chromatin, maintains a sustained H3 phosphorylation even in late mitosis (**2**). **3** A molecular gradient is also acting to control the reassembly of the nuclear pore complexes (NPC) during mitotic exit. Importin β and NPC start reassembly around the chromatin from the poleward side

Within the cell, spatial information is pivotal for the execution of several processes. During mitotic exit, although the time direction is dictated by the decline in CDK activity and the affinity of the relevant phosphatases to substrates, the same substrate can be dephosphorylated at different times according to its localisation in the cell. A well-known example is the dephosphorylation of histone H3. Here, dephosphorylation starts occurring at the pole side of the chromosomes and gradually proceeds towards the telomeres as the chromatids move further away from the midzone and achieve their maximum compaction in telophase (Fig. 3, 2–3). At the same time, the reformation of nuclear structure, in organisms where the nuclear envelope is remodelled during mitosis, can occur with a distinct pattern e.g., the nuclear pores assembly around the chromatin starts from the pole side of the segregating chromosomes (Fig. 3, 3).

After CDK1 inactivation, other kinases such as Polo and Aurora B that are somehow dependent on CDKs (Nigg 2001a, b) still maintain their activity for a prolonged period during mitotic exit since they are necessary for specific late mitotic events and their localization is targeted to specific subcellular structures.

As a general principle, both the phosphatase and the kinase reactions need to be taken into account to predict the phosphorylation equilibrium of a substrate; the persistence of a substrate in proximity to the kinase source will ultimately tip the balance toward phosphorylation even if the phosphatases are fully active. These observations led to the formulation of the gradient hypothesis during mitotic exit. The simplest model for a gradient requires (1) a protein that exists in the de-phospho (D) and phospho-state (P), (2) a kinase that converts the protein from D to P and (3) a phosphatase that converts the protein from P to D. If either the kinase or the phosphatase is bound to cellular structures, then a gradient of phosphorylation can be established within the cell. This system works both for freely diffusible substrates and for substrates where the diffusion coefficient is dictated by other cellular movements e.g., the movement of the chromatids towards the poles. This model has been shown to exist for Aurora B during mitotic exit where the kinase phosphorylates chromosome substrates that are dephosphorylated by PP1, a chromosome-associated phosphatase at anaphase (Fuller et al. 2008).

The gradient distribution of Aurora B substrates in anaphase seems to be quite a common feature, and it has been reported for a growing number of substrates such as histone H3S10, H3S28, class IIa HDACs and EB3 (S176) (Guise et al. 2012). The localization of the gradient is extremely important in the coordination of events that leads to the establishment of a functional G1 nucleus; for example, it prevents chromosome decondensation and nuclear envelope reassembly (NER) until effective separation of sister chromatids is achieved thus acting as a mechanism to reduce the occurrence of micronuclei after mitosis (Afonso et al. 2014). While the existence of an Aurora B gradient in mammalian cells is well documented, very little is known if Polo-like kinase is capable of such spatial control. In theory, it should work for this kinase as well since its localization is compartmentalised during mitosis; however, previous work using a PLK sensor did not reveal a spatial phosphorylation pattern in anaphase (Fuller et al. 2008). The different mechanism of action of the two kinases might be the reason for the different spatial behaviour.

### Mechanical forces

So far, we have a general understanding of how the transition from mitosis into the new G1 nucleus is temporally (molecular clocks) and spatially (molecular gradients) regulated. However, just the simple observation of a mammalian cell dividing prompts us to consider that the mechanics of the process may play important roles as well. The movement towards the poles, the invagination and cleavage of the furrow, the spreading of the cells all generate local tensions. Moreover, the reformation of the nuclear membrane and intranuclear structures may well exert a mechanical role in the establishment of the chromosome territories and chromatin organisation in a few hours window after mitosis.

This aspect is not yet well studied but there are indications that mechanical forces are important players to be considered in the process. Recent work from Funabiki’s laboratory has shown that drastically changing microtubule dynamics during pronuclear reassembly in Xenopus egg extracts causes the appearance of distorted and irregularly shaped nuclei. The chromatin-associated protein Dppa2 (development pluripotency associated 2) appears to be the regulator of this process. The importance of this mechanical clue in the formation of the G1 nucleus is revealed also by the fact that these nuclei present a delayed and disorganised DNA replication (Xue et al. 2013). It would be interesting to assess if this mechanism is also in place in somatic cells and to which extent it affects gene expression and chromatin organisation. This first study seems to suggest that physical interactions between the anaphase/telophase chromatin and the cytoskeleton have major implications in the re-establishment of a functional G1 nucleus. The observation is not surprising considering that after division there are physical connections between the nuclear skeleton and the cytoplasm via the linker of nucleoskeleton and cytoskeleton (LINC) complex. This complex is involved in actin-dependent nuclear movement in polarising fibroblasts (Luxton et al. 2010) and microtubule and dynein-mediated movement of nuclei in migrating neurons and developing photoreceptor cells (Zhang et al. 2009) (Yu et al. 2011). Recently, this complex has also been implicated in very rapid mechano-chemical signalling to the nucleus (Isermann and Lammerding 2013) (Guilluy et al. 2014). The signalling pathway that is triggered by mechanical forces includes the recruitment of lamin A/C to the LINC complex and phosphorylation of emerin. We can therefore imagine that, in a similar manner, changes in the mechanics of cytokinesis could produce a different recruitment of lamina-associated complexes that ultimately will influence the establishment of a chromatin landscape in G1.

Among the forces that could play an important role in shaping the G1 nucleus after mitosis are dynein-mediated pulling forces on astral microtubules and contraction of the equatorial actomyosin ring coupled to the polar cortex relaxation. These mechanisms have been shown to be essential for giving the overall power in separating sister chromatids during anaphase (Zheng et al. 2014) and to constrain the anaphase spindle rocking (Rankin and Wordeman 2010) and cleavage furrow positioning (Sedzinski et al. 2011), respectively.

Moreover, NuMa has also been shown to have specific cortical receptors, which allows recruitment of dynein/dynactin to the cell cortex during anaphase. These interactions only occur after dephosphorylation of NuMa in anaphase and are important possibly to increase the cortical pulling forces necessary to ensure proper spindle elongation (Kiyomitsu and Cheeseman 2013). However, more studies are required to understand what (if any) their contribution toward the establishment of the G1 nucleus is.

## Conclusions and future directions

Recent developments in understanding how mitotic exit is driven and regulated have shed light on the main players of this complicated process. However, several aspects remain elusive. From the chromatin point of view, re-establishing the epigenetic status, configuring for binding of chromatin proteins and transcription itself are some of the challenges. The list of known phosphatases and targeting subunits is increasing but we are well behind with the identification of their crucial substrates. Repo-Man bound to PP1 has been found to aid in the dephosphorylation of T3/S10/S28; however, other post translational modifications (namely other than phosphorylations) might be involved in the condensation and silencing of mitotic chromatin. Similarly, ejection of chromatin binding proteins through phosphorylation events might just be the tip of the iceberg with regards to modifications that will need to be reverted for re-establishment of chromatin environments in G1. Reassembly of the nuclear envelope implicates a consortium of synchronised events to ensure the rim formation, nuclear pore assembly and import functions. The physics behind the pushing and pulling forces of a cell undergoing mitosis also remains far from understood. How kinases/phosphatases timely regulate the breakdown of the nuclear envelope, movement of chromatin to distal parts, and even the organisation of chromatin upon the formation of the G1 nucleus remains to be elucidated.

The cooperation between the development of new imaging technologies, single cell analyses and mathematical modelling will be essential to understand in the four-dimensional space the pivotal factors which regulate the formation of a structure required for efficient regulation of the nucleus after mitosis is over.
